# RETRACTION: Downregulation of SUMO2 inhibits hepatocellular carcinoma cell proliferation, migration and invasion

**DOI:** 10.1002/2211-5463.70248

**Published:** 2026-04-15

## Abstract

**RETRACTION:** J. Chen, C. Chen, Y. Lin, Y. Su, X. Yu, Y. Jiang, Z. Chen, SY. Ke, SZ. Lin, L. Chen, Z. Zhang, and T. Zhang, “Downregulation of SUMO2 inhibits hepatocellular carcinoma cell proliferation, migration and invasion,” *FEBS Open Bio* 11, no. 6 (2021): 1771–1784. https://doi.org/10.1002/2211-5463.13173.

The above article, published online on 14 May 2021 in Wiley Online Library (wileyonlinelibrary.com), has been retracted by agreement between the journal Editor‐in‐Chief, Miguel De la Rosa; the Federation of European Biochemical Societies; and John Wiley & Sons Ltd. A third party reported that images in the Bel‐7404 cell migration assay in Figure 5A contained overlapping duplicated sections and that the SMMC‐7721 cell invasion assay in Figure 5C also contained overlapping duplicated sections. Further investigation by the editors and the publisher found additional overlapping duplicated sections in the SMMC‐7721 cell migration assay images in Figure 5A and potential duplication of a portion of the GAPDH band between the Bel‐7404 image in Figure 4B and Bel‐7404 image in Figure 5F.

The authors responded to an inquiry by the publisher. However, the editors found that the explanation and raw data provided by the authors did not adequately address the concerns. The evidence of image overlap and duplication in several figures has compromised the editor's confidence in the conclusions presented in the manuscript. As a result, the Editor‐in‐Chief, FEBS Press, and John Wiley and Sons Ltd. have determined that a retraction is necessary. The authors were informed about the retraction.

**RETRACTION:** J. Chen, C. Chen, Y. Lin, Y. Su, X. Yu, Y. Jiang, Z. Chen, SY. Ke, SZ. Lin, L. Chen, Z. Zhang, and T. Zhang, “Downregulation of SUMO2 inhibits hepatocellular carcinoma cell proliferation, migration and invasion,” *FEBS Open Bio* 11, no. 6 (2021): 1771–1784. https://doi.org/10.1002/2211-5463.13173.

The above article, published online on 14 May 2021 in Wiley Online Library (wileyonlinelibrary.com), has been retracted by agreement between the journal Editor‐in‐Chief, Miguel De la Rosa; the Federation of European Biochemical Societies; and John Wiley & Sons Ltd. A third party reported that images in the Bel‐7404 cell migration assay in Figure 5A contained overlapping duplicated sections and that the SMMC‐7721 cell invasion assay in Figure 5C also contained overlapping duplicated sections. Further investigation by the editors and the publisher found additional overlapping duplicated sections in the SMMC‐7721 cell migration assay images in Figure 5A and potential duplication of a portion of the GAPDH band between the Bel‐7404 image in Figure 4B and Bel‐7404 image in Figure 5F.

The authors responded to an inquiry by the publisher. However, the editors found that the explanation and raw data provided by the authors did not adequately address the concerns. The evidence of image overlap and duplication in several figures has compromised the editor's confidence in the conclusions presented in the manuscript. As a result, the Editor‐in‐Chief, FEBS Press, and John Wiley and Sons Ltd. have determined that a retraction is necessary. The authors were informed about the retraction.

